# The Emerging and Diverse Roles of Src-Like Adaptor Proteins in Health and Disease

**DOI:** 10.1155/2015/952536

**Published:** 2015-08-03

**Authors:** Nikolett Marton, Eszter Baricza, Barbara Érsek, Edit I. Buzás, György Nagy

**Affiliations:** ^1^Department of Genetics, Cell- and Immunobiology, Semmelweis University, 4 Nagyvárad Square, Budapest 1089, Hungary; ^2^MTA-SE “Lendület” Experimental and Translational Immunomics Research Group, 4 Nagyvárad Square, Budapest 1089, Hungary; ^3^Department of Rheumatology, Faculty of Medicine, Semmelweis University, 4 Frankel Leó Street, Budapest 1023, Hungary

## Abstract

Although Src-like adaptor proteins (SLAP-1 and SLAP-2) were mainly studied in lymphocytes, where they act as negative regulators and provide fine control of receptor signaling, recently, several other functions of these proteins were discovered. In addition to the well-characterized immunoregulatory functions, SLAP proteins appear to have an essential role in the pathogenesis of type I hypersensitivity, osteoporosis, and numerous malignant diseases. Both adaptor proteins are expressed in a wide variety of tissues, where they have mostly inhibitory effects on multiple intracellular signaling pathways. In this review, we summarize the diverse effects of SLAP proteins.

## 1. Introduction

**Figure 1:**
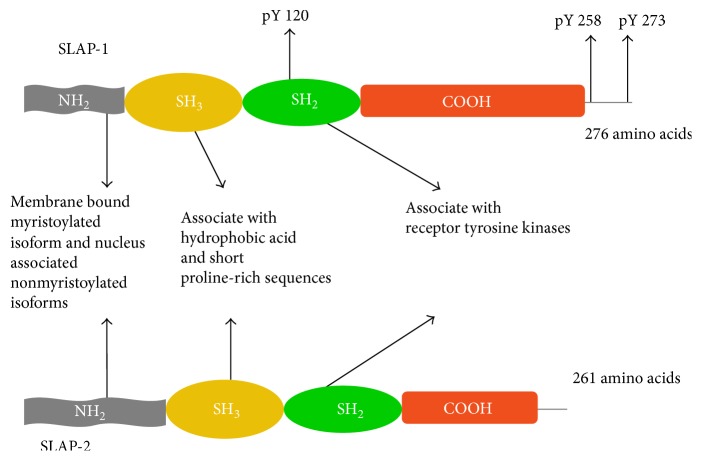
The schematic structure of SLAP molecules. SLAP-1 and SLAP-2 contain unique carboxy-terminal sequences, common SH2-SH3 domains and amino-terminals which exist in myristoylated and nonmyristoylated isoforms. SH2 domains help molecules to bind to phosphorylated tyrosine containing epitopes. The SH3 domains connect the proline and hydrophobic amino acid containing molecules.

Signal transducing adaptor proteins are a group of intracellular and transmembrane molecules which are crucial supplementary factors of signaling pathways. They mediate interactions between different molecules and contribute to the formation of signaling complexes. Adaptor proteins lack enzymatic activity and interaction domains enable them to connect with other molecules (e.g., proteins, lipids). Src-like adaptor protein 1 (SLAP-1) was cloned in a yeast two-hybrid screen with the cytoplasmic domain of the receptor tyrosine kinase ephrin type-A receptor 2 as decoy [1, 2]. SLAP proteins are not identical in size; SLAP-1 (or often quoted as SLAP) contains 276 amino acids while SLAP-2 consists of 261 amino acids (Figure 1). SLAP-1 and SLAP-2 contain common SH2-SH3 domains. SH2 domains allow proteins to dock to phosphorylated tyrosine containing epitopes. SH3 domains bind to hydrophobic amino acid and proline rich molecules. SH domains of SLAP-2 form continuous *β*-sheet that stretches the modular domains [3].

SLAP proteins were named after Src family kinases which have the same SH sequences. Src protein tyrosine kinases (PTKs) are protooncogenes that play a role in cell proliferation, survival, and morphology. Unlike Src PTKs, SLAP molecules do not have tyrosine kinase domains. In addition to the myristoylated amino-terminal and SH domains, SLAP family members contain unique carboxy-terminal sequences as well. SLAP-1 has a longer carboxy-tail than SLAP-2, but the N-terminal is longer in SLAP-2. Myristoylated part of the SLAP promotes the association with membranes, while isoforms without the myristoylated N-terminal are located in the nucleus (Figure 1). Human SLAP is coded by a 64 kb intron of the thyroglobulin gene, on chromosome 8q24.23 in the candidate territory for a recessive demyelinating neuropathy. Sequence analysis could not find any mutations suggesting that this gene is not responsible for the disease [4]. Human SLA2 gene is located on 20q11.23 [1].

**Figure 2:**
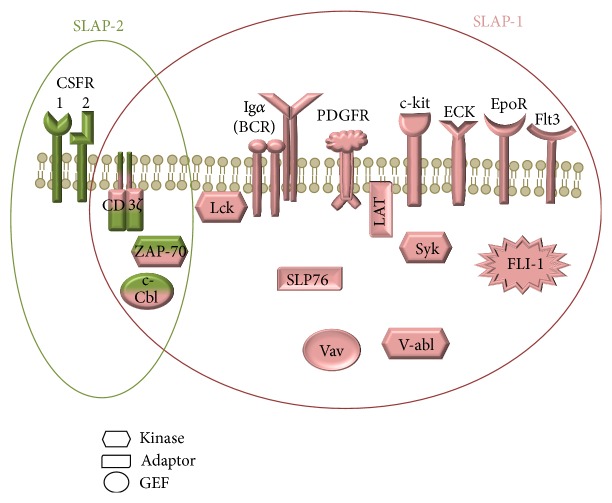
The interaction partners of SLAP-1 (pink) and SLAP-2 (green). Several proteins have been reported to interact with SLAP-1 molecule: c-Cbl, CD3 *ζ* chain, ECK, EpoR, Ig*α*, LAT, Lck, PDGFR, SLP-76, Syk, v-abl, Vav (protooncogene vav), ZAP70, c-kit, Flt3, and FLI. SLAP-2 interacts with c-Cbl, CD3 *ζ* chain, CSFR, and ZAP70.

Human SLAP-1 and SLAP-2 molecules show sequence homology similarly to mouse SLAP proteins. SH domains are the most similar parts of the two SLAP molecules, in which the sequence homology is 59%, while the N-terminals are the most different parts; they have only 19% identity. SLAP-2 has a shorter alternative splice variant, called SLAP-2v. This isoform contains only 210 amino acids due to the deletion of 50 bp from exon 6, which results an alternative reading frame. The splicing variant molecule does not have any c-Cbl interacting site. The biological relevance of SLAP-2v is yet unknown [5, 6]. Although expression of SLAP-1 and SLAP-2 mRNAs has been most extensively studied in lymphocytes, they are also expressed by numerous human and murine tissues and cell lines [1, 2, 7–12] (Table 1). Several proteins have been reported to interact with SLAP-1 and SLAP-2 [5, 8, 9, 13–15] (Figure 2). SLAPs are involved in a broad range of cellular processes, for example, lymphocyte development, neuronal excitotoxicity and platelet activation. SLAP molecules may participate in several pathological conditions of the immune system as well. In the present review, we will discuss the role of both SLAP proteins in different cell types and overview our current understanding regarding their relevance in pathological conditions.

## 2. SLAP Proteins in Immune Cells

### 2.1. SLAP-1 in T Lymphocytes

SLAP proteins were extensively investigated in lymphocytes where they are strongly expressed. SLAP-1 is involved in the regulation of the TCR signal transduction pathway. TCR consists of a ligand binding *αβ* heterodimer and the CD3 complex that includes the *γε* and *δε* heterodimers and the *ζζ* homodimer. The expression of the *ζ*-chains is about the 10% of the other subunits of the TCR complex. SLAP-1 may interact with many molecules involved in the TCR signal transduction (ZAP-70, Syk, LAT, CD3 *ζ*-chain, Vav, and Lck) through the SH2 domain [9, 16].

**Figure 3:**
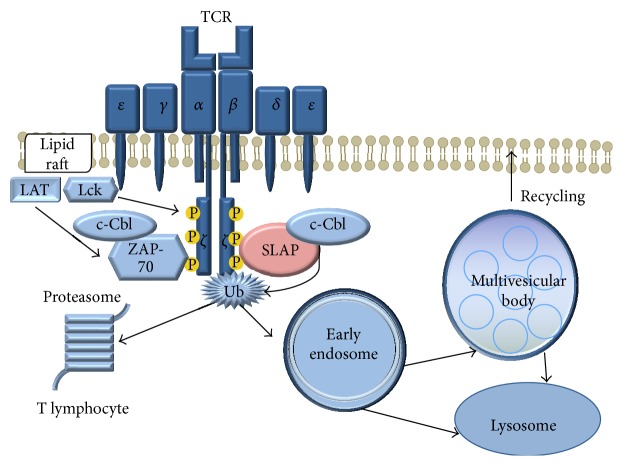
The role of SLAP in T lymphocytes.

SLAP-1 reduces the production of IL-2 and the transcription of NFATc1 and AP-1, thereafter functions as a negative regulator of the TCR signaling; both SH2 and SH3 domains are necessary for this effect. However, upon ionomycin or PMA activation of lymphocytes, these inhibitory effects are absent suggesting that SLAP-1 regulates the proximal part of the TCR pathway [7]. SLAP may associate with the N-terminal of the E3 ubiquitin ligase c-Cbl in a tyrosine phosphorylation independent way [16]. The simultaneous expression of SLAP-1 and c-Cbl promotes the ubiquitination and degradation of the *ζ*-chain, consequently enhancing the recycling and preventing the accumulation of the receptor complexes [17]. By contrast, similarly to SLAP −/− lymphocytes, c-Cbl −/− lymphocytes overexpress the *ζ*-chain due to its abolished degradation [18]. For successful operation, SLAP-1 requires the phosphorylation of the cytoplasmic domains of the TCR *ζ*-chains and the activation of Lck, but not of ZAP70 [19]. It has been shown that the phosphorylated proportion of the *ζ*-chains is slightly traceable in Lck −/− lymphocytes [20, 21]. SLAP colocalizes with early endosomes according to confocal microscopy images [7] (Figure 3). It is noteworthy that downregulation of *ζ*-chain of T-cells has been observed in many pathological conditions including rheumatoid arthritis (RA), systemic lupus erythematosus (SLE), human immunodeficiency virus (HIV) infection, and various cancers [22–28].

Thymic selection is essential in shaping the peripheral T cell repertoire. The central role of SLAP in lymphocyte development has been also described. The fate of the early lymphocytes depends on the TCR-mediated signals: both too strong and too week signals through the TCR lead to cell death (during positive and negative selections, resp.). The expression of SLAP is strictly regulated during the thymic development of lymphocytes. It is expressed at low levels in CD4− CD8− cells and at high levels in CD4+ CD8+ thymocytes. SLAP as a regulator of the TCR expression plays a pivotal role in the downregulation of TCR complexes of the developing lymphocytes. Enhanced positive selection was observed in SLAP deficient mice. Moreover, the apoptosis of ZAP70 −/− cells was inhibited in the absence of SLAP [29]. It has been reported that the proline rich-sequence (PRS) of CD3*ε* act together with SLAP in the regulation of TCR expression in CD4+ CD8+ thymocytes. CD3*ε* PRS deficient cells were unable to degrade the *ζ*-chain [30]. In double positive thymocytes, the chains of the TCR complex are constitutively ubiquitinated, but the ubiquitination is absent in mature cells. It has been shown that CD3*ε* PRS, Lck, c-Cbl, and SLAP are required for the ubiquitination and degradation of the *ζ*-chains. In the absence of ubiquitination, both the lysosomal sequestration and degradation are failed, and TCR chains are upregulated in CD4+ CD8+ lymphocytes. In addition, modified TCR complex ubiquitination influences the formation of the immunological synapse and alters the selection of the immature cells [31]. The lack of SLAP increases the avidity of the TCR which leads to the negative selection of antigen specific CD8+ cells. All these data suggest that SLAP plays a major role in the TCR repertoire configuration [32].

### 2.2. SLAP-1 in B Lymphocytes

**Figure 4:**
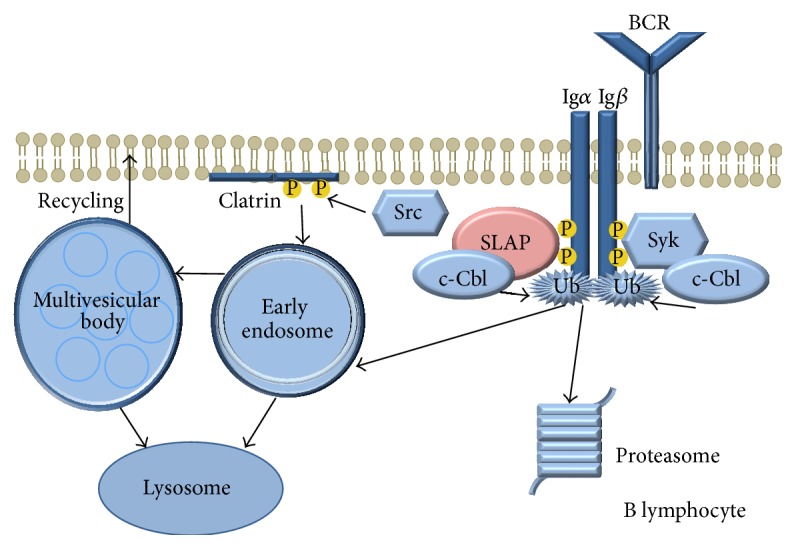
The role of SLAP in B lymphocytes.

SLAP is associated with c-Cbl in B lymphocytes, leading to BCR recycling [33] (Figure 4). SLAP deficiency increases the BCR levels of immature B cells in HEL-specific MD4- transgenic mice, which is a frequently used model to study naïve B lymphocytes. In this model the upregulated receptor complex levels lead to increased signal transduction. SLAP deficient mice have an increased number of splenic B cells, but the surface expression of BCR and IgM of mature lymphocytes is decreased. Furthermore, the activation induced calcium flux is diminished in SLAP KO B cells. In virtue of the previous data SLAP regulates the level of BCR s which is essential for the adequate development and function of B cells [34].

### 2.3. SLAP-2 in T and B Lymphocytes

Similarly to SLAP-1, SLAP-2 also has an effect on lymphocyte receptor signalization. SLAP-2 has a negative regulatory role on the antigen receptor signaling of T and B lymphocytes. The overexpression of SLAP-2 reduces the surface levels of CD3 [5]. The overexpression of SLAP-1 and SLAP-2 inhibits the upregulation of CD69 after antigen receptor cross-linking. CD69 is an inducible cell surface glycoprotein, upregulated during lymphocyte activation. SLAP-2 suppresses the antigen binding induced calcium influx in T (Jurkat) and B (BJAB) cell lines. SLAP-2 reduces the CD69 expression in Jurkat cells (61%) more significantly than in BJAB cells (28%). Similarly to SLAP-1, SLAP-2 does not impair the ionomycin and PMA induced signalization [35]. SLAP-2 is associated with ubiquitin ligase c-Cbl similarly to SLAP-1, deletion of the carboxy-terminal of SLAP-2 was reported to inhibit this connection [35]. In activated Jurkat cells SLAP-2 binds to c-Cbl, ZAP-70, and CD3 *ζ* in a phosphorylation independent manner [1]. The coexpression of SLAP-2 and ZAP-70 or Syk in T-cell lines lead to the degradation of both kinases. Thus, SLAP-2 induces the c-Cbl dependent degradation of tyrosine kinases and downregulates CD3 expression.

### 2.4. SLAP Molecules in Monocytes and Dendritic Cells

**Figure 5:**
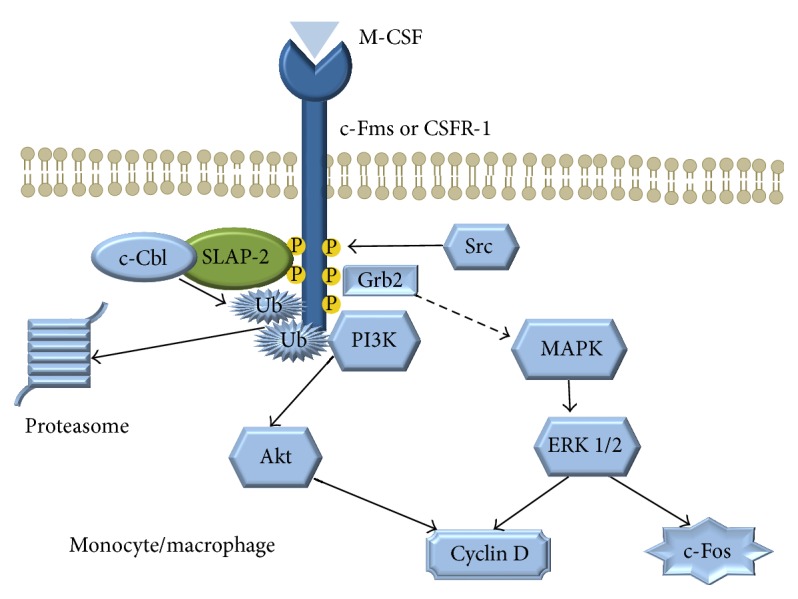
The role of SLAP-2 in monocytes/macrophages.

SLAP-2 is expressed in human monocytes and bone marrow cells [1, 35], but not in CSF1 independent monocyte cell lines such as RAW264.7 [5]. It was shown that SLAP-2 may bind to both c-Fms and c-Cbl, and several domains of SLAP-2 are involved in this interaction (Figure 5). The overexpression of SLAP-2 in murine bone marrow reduced the M-CSF-induced tyrosine phosphorylation [36]. SLAP-2 downregulates the c-Fms signaling through a c-Cbl dependent internalization and degradation of the CSF-1R, providing a negative feedback of the M-CSF pathway [37]. In addition, c-Fms stimulation induces the phosphorylation of the serine rich N-terminal domain of the SLAP-2 by a JNK dependent pathway [36].

The activation-induced GM-CSFR downregulation is attenuated in SLAP-1 and SLAP-2 deficient bone marrow dendritic cells (BM-DC), which is associated with enhanced MAP/Erk and Akt pathways upon GM-CSF stimulation. The inhibited activation through GM-CSFR impairs the bone marrow derived dendritic cell maturation. SLAP-1 and SLAP-2 deficient BM-DC cells produce less IL-12 and TNF-*α* upon lipopolysaccharide (LPS) stimulation and induce significantly less IFN-*γ* secretion of T-lymphocytes than the wild type cells [38]. These data suggest that SLAP proteins are necessary for monocyte and dendritic cell maturation and activation.

### 2.5. SLAP Proteins in Mast Cells

SLAP has a prominent role in the regulation of intracellular signal transduction in mast cells. SLAP-specific small interfering RNAs inhibit the effect of dexamethasone on the phosphorylation of PLC *γ*, LAT, Syk, and ERK [39]. Actinomycin D inhibits the transcription of SLAP following dexamethason treatment, suggesting that the upregulation for SLAP-1 upon glucocorticoid treatment occurs at the transcriptional level. Glucocorticoid receptor activation is required for the elevation of the SLAP transcription [40]. SLAP-1 plays a role in the negative regulation of antigen-stimulated mast cells as well. Upon antigen stimulation of the RBL-2H3 mast cell line, elevated transcription of the SLA gene and upregulation of the SLAP-1 (but not SLAP-2) protein were reported. An increased amount of SLAP-1 was detected after 60 minutes stimulation and it reached the maximum after 2-3 hours. By contrast, after silencing of SLAP, increased IL-3 and MCP-1 production was detected. Knock-down of SLAP by using siRNA increases the expression of Fc*ε*RI [39]. Thus, SLAP appears to be a crucial regulator of mast cell function.

## 3. The Function of SLAP Molecules in Other Cell Types

### 3.1. SLAP-1 in Osteoclasts

Osteoclasts play a fundamental role in the pathogenesis of osteoporosis. Inhibiting the development and activation of osteoclasts is currently the gold standard therapeutic strategy in this disease. Although today several antiosteoporotic drugs are widely used in the clinical practice, the effect of these medications on increasing bone density and strength is moderate. There is an inverse correlation between both SLAP-1 and SLAP-2 and the tartrate-resistant acid phosphatase (osteoclastogenesis marker) mRNA expression. The level of osteoclast-specific protein mRNAs (e.g., catepsin K and MMP-9) are elevated in the SLAP-1 −/− preosteoclasts. SLAP-1 has an inhibitory effect on the MAP kinase pathway which is initiated with the binding of M-CSF to its receptor c-Fms. This receptor is a tyrosine kinase transmembrane protein which accumulates in lipid rafts where it associates with SLAP-1. The lack of SLAP enhances osteoclastogenesis without changing the resorptive function of the individual cells [41]. In addition, SLAP-1 deficiency increases the apoptosis of the mature polykariotic osteoclasts without altering the viability of the precursors. According to our current understanding, SLAP-1 has a regulatory effect on osteoclastogenesis and mature cell survival through the M-CSF pathway [41].

### 3.2. SLAP Molecules in Platelets and Fibroblasts

SLAP-2 is expressed in human platelets and may associate with Syk, c-Cbl, and LAT. After its activation, SLAP-2 promotes Syk and c-Cbl to approach their substrates. SLAP-2 inhibits glycoprotein VI (GPVI) initiated signal transduction of platelets through the connection with c-Cbl [42]. This inhibitory effect on thrombocyte activation is similar to those described previously in lymphocytes [1, 5].

SLAP may also associate with platelet derived growth factor receptor (PDGFR) in NIH3T3 mouse embryonic fibroblast cell line. Overexpression of SLAP in NIH3T3 cells inhibits PDGF-induced mitogenesis [43] suggesting that SLAP is a negative regulator of growth factor initiated signaling.

### 3.3. SLAP-1 in Neurons

Although in embryonic rat telencephalon sections SLAP-1 mRNA is absent in migrating neurons, it is highly and selectively expressed in neurons which have reached their final location. SLAP-1 has a characteristic expression pattern during the development of the cortex. It is expressed mostly by deeper stratum cells especially by pyramid cells. Pyramid cells are typically located in the deeper layers of the cortex, and they have connection with the subcortical areas. It is not yet clear whether SLAP-1 plays a role in the axon guidance during development but apparently has a remarkable expression. There is also a possible interaction between SLAP-1 and EphA receptors [44].

Western blot analysis and immunolabeling of rat brain extract showed that SLAP-1 is located in the postsynaptic membrane. SLAP-1 has an association with EphAs and NMDARs have a connection with EphBs [44, 45]. EphB may excite the enlistment of EphAs and promote the recruitment of SLAP-1 and NMDARs. Despite the analogous localization, SLAP-1 does not affect the baseline activity of NMDARs through a Src-dependent stimulation of the receptors. SLAP-1 may have a role in the negative feedback which regulates the number of the NMDARs and prevent excitotoxicity. This effect of SLAP-1 is based on the proteasomal degradation of redundant NMDARs in neurons [46]. The described role of SLAP in neurons seems to be essential for the adequate neuronal functions.

## 4. SLAP in Pathological Conditions

### 4.1. The Role of SLAP in Rheumatoid Arthritis

Rheumatoid arthritis (RA) is a common autoimmune disease that is associated with progressive disability and systemic complications. Symmetric synovial inflammation of multiple joints, especially the small joints of the hands and the feet, is characteristic for the disease. The ongoing inflammation leads to cartilage destruction, bone erosions, and subsequent joint deformities. Although the current treatment strategy, especially the use of biologicals, improved largely the outcome of the disease, still only a small portion of the patients are in sustained remission and systemic complications (including cardiovascular risk) still represent a significant challenge. TNF-*α* plays an essential role in the pathogenesis of RA through promotion of angiogenesis, suppression of regulatory T-cell activation, and cytokine and chemokine expression.

According to the recently published data of our research group, TNF-*α* treatment downregulates the expression of *ζ*-chain of CD4 T-lymphocytes reversibly and selectively in a dose dependent way. Decreased *ζ*-chain expression leads to the hyporesponsiveness of T-cells. TNF-*α* induces the expression of SLAP which promotes the proteasomal but not the lysosomal degradation of the *ζ*-chain. Silencing SLAP with short interfering RNAs inhibits the TNF-*α* induced *ζ*-chain degradation. TNF-*α* treatment does not alter the SLAP mRNA level, suggesting that TNF-*α* controls SLAP activity through miRNA mediated posttranscriptional silencing. CD4+ T-lymphocytes isolated from RA patients expressed more than 2-fold higher SLAP levels than the T-cells of healthy donors. TNF-*α* treatment enhances the expression of SLAP in the CD4+ T-lymphocytes of healthy donors and DMARD treated RA patients but does not alter the expression of CD4+ T-cells isolated from biological DMARD (etanercept, certolizumab pegol) treated patients [47].

A spontaneous mutation in ZAP70 protein uncoupled proximal TCR signal transduction and led to severe symmetrical arthritis in SKG mice upon exposure to zymosan. According to recently published data, SLAP deficiency dramatically reduced both the incidence and severity of zymosan-induced chronic autoimmune arthritis in SKG mice [48]. The protective role of SLAP deficiency was associated with the increased number of regulatory T-cells and decreased amount of Th17 cells [48].

### 4.2. The Role of SLAP in Malignant Diseases and in Erythropoiesis

SLAP expression is increased in several cancers including chronic myeloid leukemia (CML), chronic lymphocytic leukemia (CLL), and prostate cancer. By contrast, its expression is decreased in acute myeloid leukemia (AML), myeloma, and colon cancer [13]. Furthermore, SLAP is associated with several oncogenic signaling pathways.

It was recently reported that the transmembrane protooncogene c-kit is degraded through a SLAP dependent pathway. SLAP binds to the WT c-kit and initiates its ubiquitylation and degradation in the proteasome. By contrast, SLAP does not have a similar regulatory effect on the oncogenic c-kit variant (c-kit D816V). Oncogenic c-kit associates with SLAP and phosphorylates it on three different tyrosines (Y120, Y258, and Y273); the phosphorylated forms of SLAP do not alter the downstream signaling of the mutant c-Kit. These data suggest that SLAP regulates WT c-kit signaling, but the oncogenic variant escapes from this negative feedback regulation [49].

The Fms like tyrosine kinase (Flt3) is a receptor tyrosine kinase, which is predominantly expressed in hematopoietic progenitor cells. SLAP associates with both the WT and mutated, oncogenic Flt3 (Flt3-ITD). Silencing of SLAP with short interfering RNAs leads to an attenuated MAPK signal pathway. After ligand stimulation, SLAP colocalizes with the Flt3-ITD and targets it for c-Cbl dependent ubiquitylation and proteasomal degradation. About 30% of patients with AML have mutation in the Flt3 gene and gain of function mutations contribute to the initiation of AML. The expression of SLAP is increased in patients with acute promyelocytic leukemia (APL) carrying Flt3-ITD mutation as compared to the Flt3-WT [13].

FLI-1 is a transcription factor, a member of the E26 transformation-specific (ETS) protein family. Furthermore, the FLI-1 locus is an integration site for the Friend murine leukemia virus, which induces erythroleukaemia in responsive mice. SLAP protein and mRNA levels are overexpressed in FLI-1 transformed erythroblasts [8]. SLAP binds to both the phosphorylated and unphosphorylated forms of the erythropoietin receptor (EpoR). SLAP expression prevents the EPO-induced differentiation, whose effect is associated with the inhibition of STAT5 activation and BCL-X upregulation. Both STAT5 and BCL-X are critical in EPO-induced signaling [8]. SLAP may mediate the erythropoiesis in this manner.

According to recently published data, SLAP is expressed in colon epithelium, but it is significantly downregulated in colorectal tumors [15]. Interestingly, silencing of SLAP promotes tumor progression, while overexpression inhibits tumor growth and invasiveness. SLAP promotes the destabilization of the Src substrate ephrin type-A receptor 2 (EphA2) in the intestinal cells. EphA2 plays a critical role in the regulation of several intracellular signal pathways which mediate cell migration, invasion, and angiogenesis. The inhibitory effect of SLAP appears to be independent from c-Cbl but has an association with the ubiquitination factor UBE4A and with the pTyr594-EphA2. These results suggest a tumor-suppressive effect of SLAP in colorectal cancers [15].

## 5. Conclusions and Future Perspectives

SLAP proteins are expressed in a variety of cell types which indicates a conserved function of these proteins. Both SLAP-1 and SLAP-2 have a prominent role in the negative regulation of several membrane bound receptors and receptor tyrosine kinases, thereafter SLAPs play a central role in the regulation of intracellular signal transduction and cell reactivity. The proper function of SLAPs is necessary for immunoreceptor repertoire configuration and helps to avoid uncontrolled cell activation, proliferation, and migration. SLAP molecules are involved in the ubiquitination of proteins, which may lead to proteasomal degradation. Selective regulation of SLAP molecules in different cell types may allow the fine control of cell activation and differentiation. Exploring the precise role of SLAP proteins will contribute to the understanding of many yet unknown physiological regulatory processes. In addition, tissue specific targeting of SLAP may provide valuable therapeutic approaches in diverse diseases including RA, osteoporosis, immunodeficiency, and different cancers.

## Figures and Tables

**Table 1:** The expression of SLAP proteins in different tissues and cell lines.

Tissue/organ	Expressed molecule	Reference	Cell line	Expressed molecule	Reference
Blood	SLAP-1, SLAP-2	[1, 2]	BaF3	SLAP-1, SLAP-2	[5]
Bone marrow	SLAP-1, SLAP-2	[9, 10]	BJAB	SLAP-2	[35]
Brain	SLAP-1	[9]	D011.10T	SLAP-2	[5]
Colon	SLAP-2	[10]	EL4	SLAP-1	[9]
Heart	SLAP-1, SLAP-2	[10]	FLI-1 transformed erythroblasts	SLAP-1	[8]
Kidney	SLAP-1	[9]	HeLa	SLAP-1	[7]
Liver	SLAP-1	[9]	HL60	SLAP-1	[12]
Lung	SLAP-1, SLAP-2	[9]	Jurkat	SLAP-1, SLAP-2	[7, 35]
Lymph nodes	SLAP-1	[7]	NB-4	SLAP-1	[12]
Pancreas	SLAP-1	[10]	NIH3T3	SLAP-1	[10]
Placenta	SLAP-1, SLAP-2	[10]	RBL-2H3	SLAP-1	[11]
Prostate	SLAP-2	[10]	U.937	SLAP-1	[12]
Skeletal muscle	SLAP-1, SLAP-2	[9, 10]			
Small intestine	SLAP-1, SLAP-2	[10]			
Skin	SLAP-1, SLAP-2	[10]			
Spleen	SLAP-1, SLAP-2	[9, 10]			
Thymus	SLAP-1, SLAP-2	[29, 30]			

## Abbreviations

AP-1: Activator protein 1
AML: Acute myeloid leukaemia
BCLXL: B-cell lymphoma extra-large transmembrane molecule
BCR: B cell receptor
BJAB: EBV-negative, Burkitt-like lymphoma
c-Cbl: E3 Ubiquitin-protein ligase casitas B-linage lymphoma protooncogene
CD: Cluster of differentiation
CD3 *ζ*-chain: T-cell receptor complex zeta chain
c-Fms: Colony stimulating factor 1 receptor
c-kit: Mast/stem cell growth factor receptor
CSF1: Colony stimulating factor 1
DMARDs: Disease modifying antirheumatic drugs
ECK: Ephrin type-A receptor 2
Eph: Ephrin type receptor
EphA2: Ephrin type-A receptor 2
EphB: Ephrin type-B receptor
Epo: Erythropoietin
EpoR: Erythropoietin receptor
ERK: Extracellular signal-related kinases
Fc*ε*RI: Fc *ε* receptor I
FLI1: Friend leukemia integration 1 transcription factor
Flt3: Fms-like tyrosine kinase 3
GM-CSFR: Granulocyte macrophage colony stimulating factor receptor
GPVI: Glycoprotein VI
HIV: Human immunodeficiency virus
Ig*α*: B-cell receptor antigen complex-associated protein alpha
IFN *γ*: Interferon *γ*
IL: Interleukin
ITD: Internal tandem duplications
JNK: c-Jun N-terminal kinase
kb: Kilobase
LAT: Linker for activation of T-cells family member
Lck: Protooncogene tyrosine-protein kinase
MAP: Microtubule associated protein
MAPK: Microtubule associated protein kinase
MCP-1: Monocyte chemotactic protein 1
M-CSF: Macrophage colony stimulating factor
MMP9: Matrix metallopeptidase 9
mRNA: Messenger ribonucleic acid
miRNA: Microribonucleic acids
NFATc: Nuclear factor of activated T-cells
NMDAR: N-methyl-D-aspartate receptor
PDGF: Platelet derived growth factor
PDGFR: Platelet derived growth factor receptor
PLC *γ*: Phospholipase C *γ*
PMA: Phorbol myristate acetate
PTK: Protein tyrosine kinase
RA: Rheumatoid arthritis
RNA: Ribonucleic acid
SH: Src homology domain
SLA: Src-like adaptor
SLAP-1: Src-like adaptor protein 1
SLAP-2: Src-like adaptor protein 2
SLP-76: SH2 domain-containing protein of 76 kDa
Src: Sarcoma
STAT5: Signal transducer and activator of transcription 5
Syk: Spleen tyrosine kinase
Th17: T helper 17
TNF-*α*: Tumor necrosis factor *α*
TCR: T-cell receptor
UBE4A: Ubiquitin conjugation factor E4A
V-abl: Tyrosine-protein kinase transforming protein Abl
Vav: Protooncogene vav
WT: Wild- type
ZAP70: Zeta-associated protein of 70 kDa.
